# Engineering Performance of Concrete Incorporated with Recycled High-Density Polyethylene (HDPE)—A Systematic Review

**DOI:** 10.3390/polym13111885

**Published:** 2021-06-06

**Authors:** Sonali Abeysinghe, Chamila Gunasekara, Chaminda Bandara, Kate Nguyen, Ranjith Dissanayake, Priyan Mendis

**Affiliations:** 1 School of Engineering, RMIT University, Melbourne, VIC 3000, Australia; S3871542@student.rmit.edu.au (S.A.); kate.nguyen@rmit.edu.au (K.N.); 2 Faculty of Engineering, University of Peradeniya, Peradeniya 20400, Sri Lanka; csbandara@eng.pdn.ac.lk (C.B.); ranjith@fulbrightmail.org (R.D.); 3 School of Engineering, University of Melbourne, Grattan Street, Parkville, VIC 3010, Australia; pamendis@unimelb.edu.au

**Keywords:** sustainability, recycled plastic, high-density polyethylene (HDPE), concrete, construction material

## Abstract

Incorporating recycled plastic waste in concrete manufacturing is one of the most ecologically and economically sustainable solutions for the rapid trends of annual plastic disposal and natural resource depletion worldwide. This paper comprehensively reviews the literature on engineering performance of recycled high-density polyethylene (HDPE) incorporated in concrete in the forms of aggregates or fiber or cementitious material. Optimum 28-days’ compressive and flexural strength of HDPE fine aggregate concrete is observed at HDPE-10 and splitting tensile strength at HDPE-5 whereas for HDPE coarse aggregate concrete, within the range of 10% to 15% of HDPE incorporation and at HDPE-15, respectively. Similarly, 28-days’ flexural and splitting tensile strength of HDPE fiber reinforced concrete is increased to an optimum of 4.9 MPa at HDPE-3 and 4.4 MPa at HDPE-3.5, respectively, and higher than the standard/plain concrete matrix (HDPE-0) in all HDPE inclusion levels. Hydrophobicity, smooth surface texture and non-reactivity of HDPE has resulted in weaker bonds between concrete matrix and HDPE and thereby reducing both mechanical and durability performances of HDPE concrete with the increase of HDPE. Overall, this is the first ever review to present and analyze the current state of the mechanical and durability performance of recycled HDPE as a sustainable construction material, hence, advancing the research into better performance and successful applications of HDPE concrete.

## 1. Introduction

About 2.01 billion tons of Municipal Solid Waste (MSW) is generated annually worldwide, and one third of MSW is openly dumped without managing in an environmentally-friendly manner [1]. Around 40% of MSW is discharged directly to landfills, 19% of it is recovered through recycling or composting and another 11% of it is incinerated [1]. With the rapid trends of urbanization, it has been predicted that 3.40 billion tons of MSW will be generated by 2050 [1]. About 12% of MSW generated are plastics, which is approximately 24.14 million tons [1]. The plastic industry began in the early 1900s in the USA [2]. During the period of 1950 to 2015, 8.3 billion tons of plastics were manufactured worldwide and 6.3 billion tons of them were discharged as waste [3]. Only 9% of plastic waste had been recycled, 12% were incinerated and the majority of 79% was discharged into landfills or openly dumped [3]. China ranks at the top in plastic manufacturing followed by Europe which accounts for 30% and 19%, respectively. Furthermore, China tops even in plastic consumption followed by Western Europe which is around 20% and 18%, respectively [4]. Plastic is one of the vastly discharged wastes to the environment which has adversely affected the wildlife, their habitats, and humans continuously over the past few decades. This emphasizes that it is high time to rethink the necessity of plastic recycling and reusing.

**Figure 1:**
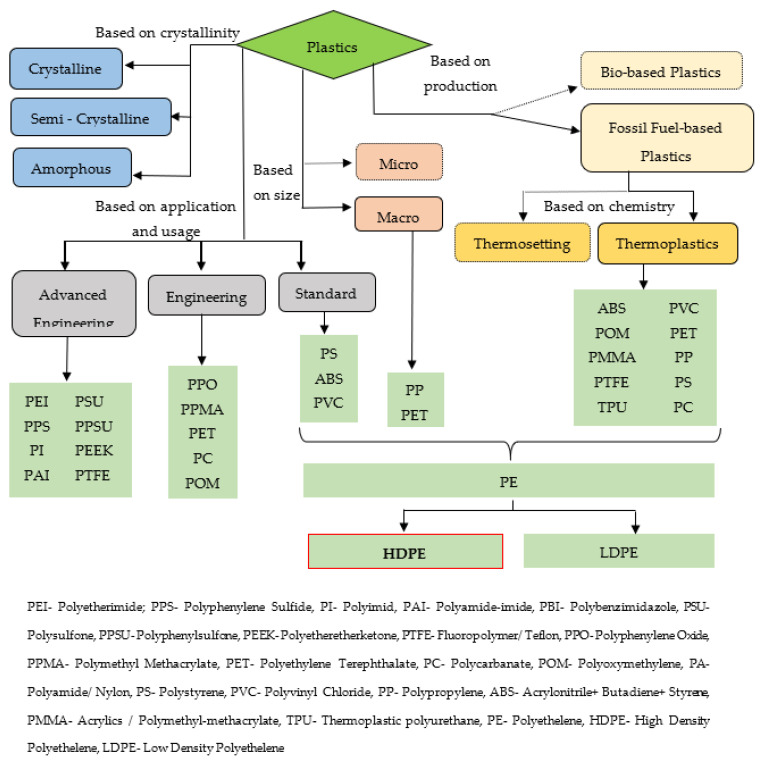
Plastic categorization.

Today, more than 30 types of primary plastics and over thousands of different secondary plastics can be found, produced by using different combinations and proportions of primary plastics [5]. Plastics can also be categorized based on many aspects as illustrated in Figure 1 [4,5]. Different plastics have different characteristics such as mechanical properties, durability resistances and diverse applications based on their compositions and chemical structures. The most commonly manufactured and applied plastics in the world are polyethylene (PE), polypropylene (PP), polyvinyl chloride (PVC), polyethylene terephthalate (PET) and polystyrene (PS) which represents 69% of the global plastic consumption [4]. Out of global plastic production, the polypropylene (PP) and low-density polyethylene (LDPE) account for 17% and 16%, respectively, followed by high-density polyethylene (HDPE) (13%) and polyphthalamide (PP&A) (13%). In addition, additives used in plastic products’ manufacturing also have a significant share in global plastic production (6%) [4]. Moreover, the use of combustible claddings in high-rises significantly increase the risk of fire spread via the external façades [6]. Urgent work on the removal of polyethylene-based claddings is necessary to ensure the safety of the occupants and buildings but also accounts for a substantial amount of PE sent to landfill.

Concrete is ranked as the topmost man-made resource utilized in the construction industry worldwide. The global aggregate requirement for concrete production accounts for about 4.5 billion tons per year which alarms the necessity of finding alternative aggregate sources required for increasing trends of concrete production [7]. Incorporating recycled plastic waste in concrete production is a sustainable approach for both disposing of plastic waste and aggregate scarcity, due to its economic and ecological advantages. Concrete composites replace various types of recycled plastics in the forms of aggregate, binder, filler or fiber reinforcement in different proportions where concrete properties are optimized [8]. PP, PET and HDPE are the most used plastics in the construction industry. However, the application and research studies on HDPE being used with concrete is very minimal compared with PP and PET.

HDPE is a thermoplastic synthetic polymer in the subset of the PE macro plastic group. PE polymer consists of neverlasting hydrocarbon chains where each carbon molecule is bound to another two carbon molecules and two hydrogen molecules. HDPE has minimal branches in its polymer chain which results to pack liner molecular chains together regularly during crystallization. As a result, semi-crystalline HDPE polymers become much more dense, rigid, and ductile with a comparatively strong bending strength between 20 to 45 MPa due to the regular packing of polymer chains [3,4,5]. HDPE also has a low density between 950 to 970 kg/m3, a better flexibility, and a high tensile strength between 20 to 32 MPa [3,4,5]. Further, HDPE is a chemically inert [3,4,5] material and its melting point is at 130 °C while ignition temperature is at 487 °C [9].

## 2. Significance of the Review

**Figure 2:**
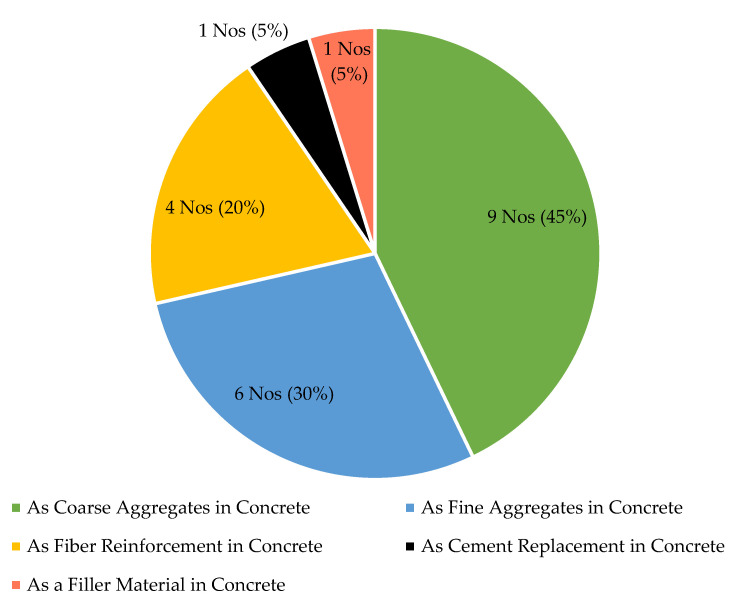
Use of HDPE as a construction material in concrete.

HDPE is the third most applied plastic type in construction preceded by PE and PET. However, a little research is reported in concrete incorporated with HDPE as a construction material, Figure 2. Moreover, there is no systematic review which was conducted by discussing existing research findings of HDPE concrete. Hence, this comprehensive review will provide the clear insight and in-depth analysis of the existing HDPE concrete performance, prevailing research gaps and future research improvements required.

## 3. Use of HDPE as an Aggregate in Concrete

### 3.1. Mechanical Properties of HDPE Fine Aggregate Concrete

**Figure 3:**
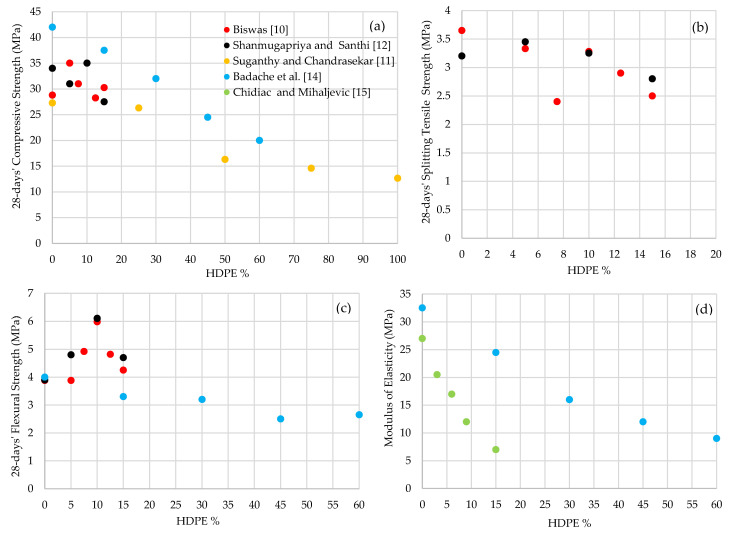
28-days’ (**a**) Compressive strength; (**b**) Splitting tensile strength; (**c**) Flexural strength and (**d**) Modulus of elasticity variation of HDPE fine aggregate concrete.

Throughout the paper, HDPE incorporated concrete is denoted in an abbreviation form, such as 10% of HDPE inclusion as HDPE-10 and 100% of HDPE inclusion as HDPE-100, etc. Table 1 and Table 2 summarizes the mechanical performances of HDPE fine aggregate concrete. It was observed that the slump (workability) is decreased from 70 mm to 30 mm when HDPE is increased from 5% to 15% while keeping the w/c ratio a constant of 0.42 [10]. In another experiment, a constant slump of 90 mm is observed with the increase of w/c ratio as 0.45 for HDPE-0, 0.50 for HDPE-25 and HDPE-50, 0.55 for HDPE-75 and 0.6 for HDPE-100 [11]. These conditions have resulted in a decrease in the compressive strength by 40.2% when HDPE is increased from 0% to 50% and by 22.3% when HDPE is increased from 50% to 100% [11]. A decreasing trend in the compressive strength of HDPE fine aggregate concrete with the increase of HDPE percentage was observed. As illustrated in Figure 3a, the 28-days’ compressive strength varied between 27.5 MPa to 42 MPa when HDPE is added from 0% to 20% [10,12,13]. Shanmugapriya and Santhi [12] observed a 3 MPa drop in compressive strength when HDPE is increased from 0% to 5%. Then the optimum compressive strength of 35 MPa was recorded at HDPE-10 [12]. Galupino et al. [13] have also observed the optimum compressive strength of 38.6 MPa at HDPE-10. Biswas [10] has obtained 35 MPa of compressive strength at HDPE-5 concrete but decreased by 19.3% when HDPE increased to 12.5%. Badache et al. [14] have observed a decreasing trend in 28-days’ compressive strength with a drop of 52.4% and 57.8% when HDPE is increased from 0% to 50% and 50% to 100%, respectively.

The 28-days’ splitting tensile strength also shows a decreasing trend with the increase of HDPE content from 0% to 15%, Figure 3b. The HDPE-5 concrete shows the highest splitting tensile strength, 3.45 MPa, which is a 31.25% increase than the standard concrete of HDPE-0 [12]. However, a drop of 18.8% is observed with the increase of HDPE from 5% to 15% [12]. In another study, it was observed a 8.7% and 27.9% of splitting tensile strength drop when HDPE is added from 0% to 5% and from 5% to 7.5%, respectively [10]. There was a tensile strength gain of 26.8% when HDPE is increased from 7.5% to 10%, however, a drop of 23.8% was noted when HDPE is increased from 10% to 15% [10].

Similar to compressive and splitting tensile strengths, the flexural strength also decreases with the increases of HDPE fine aggregate percentage, Figure 3c. It is interesting to note that the flexural strength of concrete incorporated with HDPE up to 15% was higher than the standard concrete [10,12]. The flexural strength has increased by 56.4% with the increase of HDPE from 0% to 10% and obtained the optimum flexural strength of 6.1 MPa at HDPE-10 [12]. Then, a slight strength drop of 1.4 MPa was observed at HDPE-15 [12]. A similar flexural strength development was observed by Biswas et al. [10] when HDPE is increased from 0% to 15%. There was no change in flexural strength in HDPE-0 and HDPE-5 concretes, and then a 54.1% strength gain was noted with the increase of HDPE from 5% to 10% and obtained optimum flexural strength of 5.98 MPa at HDPE-10 [10]. A drop of 28.9% was observed, when HDPE is added from 10% to 15% [10].

The modulus of elasticity is decreased with the increase of HDPE percentage, Table 2. According to Figure 3d, there was a considerable elasticity modulus decrease, 74%, when HDPE content increases up to 15% [14,15]. It is observed that the density of the HDPE fine aggregate concrete is decreased with the increase of recycled HDPE content, Table 2. The density of HDPE fine aggregate concrete has varied from 2165 kg/m^3^ to 1825 kg/m^3^, when HDPE increases from 0% to 15% [15]. Badache et al. [14] have observed a 20% density reduction with the increase of HDPE from 0% to 60% and obtained a density of 1760 kg/m3 at HDPE-60.

**Table 1:** Mix design and compressive strength properties of HDPE fine aggregate concrete.

Reference	Mix Design									Mechanical Properties							
	Cement (kg/m^3^)	HDPE %	Sand %	HDPE (kg/m^3^)	Sand (kg/m^3^)	Coarse Aggregates (kg/m^3^)	Water (kg/m^3^)	W/C Ratio	Admixtures/Superplasticizer (w%)	Compressive Strength (MPa)							
										3 days	7 days	14 days	28 days	90 days	120 days	160 days	180 days
[10]	315	0	100	0	892.2	1285.5	132.3	0.42	-	-	17.77	23.09	28.8	-	-	-	-
[13]	-	0	100	-	-	-	-	-	-	-	29.65	33.49	34.2	-	34.38	-	-
[12]	320	0	100	-	848.6	1286.5	134.4	0.42	-	-	24	28	34	-	-	-	-
[11]	-	0	100	-	-	-	-	0.45	-	-	15.55	24.75	27.26	-	-	-	-
[15]	118 kg	0	100	-	841 kg	522 kg	32 l	-	-	19	-	-	-	-	-	32.5	-
[14]	400	0	100	-	-	-	200	0.5	0.8	-	34.5	-	42	43	-	-	43
[15]	118 kg	3	97	-	816 kg	522 kg	30 l	-	-	18.5	-	-	-	-	-	25	-
[10]	315	5	95	44.6	847.6	1285.5	132.3	0.42	-	-	23	27.8	35	-	-	-	-
[13]	-	5	95	-	-	-	-	-	-	-	15.69	18.41	28.57	-	28.63	-	-
[12]	320	5	95	-	806.2	1286.5	134.4	0.42	-	-	22	24.5	31	-	-	-	-
[15]	118 kg	6	94	-	790 kg	522 kg	30 l	-	-	14	-	-	-	-	-	19	-
[10]	315	7.5	92.5	65.3	805.2	1285.5	132.3	0.42	-	-	21	25	31	-	-	-	-
[15]	118 kg	9	91	-	765 kg	522 kg	30 l	-	-	12	-	-	-	-	-	15.5	-
[10]	315	10	90	84.8	762.8	1285.5	132.3	0.42	-	-	24.2	26.8	35.01	-	-	-	-
[13]	-	10	90	-	-	-	-	-	-	-	23.69	34.87	38.6	-	38.89	-	-
[12]	320	10	90	-	763.8	1286.5	134.4	0.42	-	-	24	27.5	35	-	-	-	-
[10]	315	12.5	87.5	103.2	722.3	1285.5	132.3	0.42	-	-	18.9	24	28.25	-	-	-	-
[10]	315	15	85	149.6	847.6	1156.86	132.3	0.42	-	-	22.9	25	30.25	-	-	-	-
[13]	-	15	85	-	-	-	-	-	-	-	23.76	27.48	20.89	-	31.32	-	-
[12]	320	15	85	-	721.3	1286.5	134.4	0.42	-	-	18	22	27.5	-	-	-	-
[14]	400	15	85	-	-	-	200	0.5	0.7	-	31.5	-	37.5	39	-	-	40
[15]	118 kg	15	85	-	715 kg	522 kg	29 l	-	-	6.5	-	-	-	-	-	8.5	-
[11]	-	25	75	-	-	-	-	0.5	-	-	12.44	23.852	26.29	-	-	-	-
[14]	400	30	70	-	-	-	200	0.5	0.6	-	27.5	-	32	36	-	-	36.5
[14]	400	45	55	-	-	-	200	0.5	0.55	-	23	-	24.5	29.5	-	-	29
[11]	-	50	50	-	-	-	393.75	0.5	-	-	11.33	14.81	16.29	-	-	-	-
[14]	400	60	40	-	-	-	200	0.5	0.5	-	18.5	-	20	25	-	-	26
[11]	-	75	25	-	-	-	433.125	0.55	-	-	9.55	13.18	14.59	-	-	-	-
[11]	-	100	0		-	-	472.5	0.6	-	-	9.1	11.55	12.66	-	-	-	-

**Table 2:** Tensile strength, elastic modulus, density, and workability properties of HDPE fine aggregate concrete.

Reference	Mechanical Properties										
	Split Tensile Strength (MPa)			Flexural Strength (MPa)					Modulus of Elasticity (GPa)	Density (Kg/m^3^)	Slump (mm)
	7 days	14 days	28 days	7 days	14 days	28 days	90 days	180 days			
[10]	2.1	2.72	3.65	2.92	3.28	3.88	-	-	-	-	-
[13]	-	-	-	-	-	-	-	-	-	-	-
[12]	1.25	2.9	3.2	2.4	3.1	3.9	-	-	-	-	-
[11]	-	-	-	-	-	-	-	-	-	-	90
[15]	-	-	-	-	-	-	-	-	27	2165	-
[14]	-	-	-	3.75	-	4	4.15	4.75	32.5	2220	-
[15]	-	-	-	-	-	-	-	-	20.5	2097	-
[10]	1.28	2.75	3.33	2.15	3.1	3.88	-	-	-	-	70
[13]	-	-	-	-	-	-	-	-	-	-	-
[12]	1.25	2.85	3.45	2.5	3.8	4.8	-	-	-	-	-
[15]	-	-	-	-	-	-	-	-	17	2022	-
[10]	1.28	1.8	2.4	2.18	2.26	4.92	-	-	-	-	65
[15]	-	-	-	-	-	-	-	-	12	1930	-
[10]	2.02	2.65	3.28	3.2	3.2	5.98	-	-	-	-	45
[13]	-	-	-	-	-	-	-	-	-	-	-
[12]	1.95	2.65	3.25	3.5	4.7	6.1	-	-	-	-	-
[10]	1.88	2.38	2.9	2.28	2.27	4.82	-	-	-	-	40
[10]	1.35	2.01	2.5	3.27	3.15	4.25	-	-	-	-	30
[13]	-	-	-	-	-	-	-	-	-	-	-
[12]	1.8	2.3	2.8	2.2	3.6	4.7	-	-	-	-	-
[14]	-	-	-	3.25	-	3.3	3.7	4.4	24.5	2120	-
[15]	-	-	-	-	-	-	-	-	7	1825	-
[11]	-	-	-	-	-	-	-	-	-	-	90
[14]	-	-	-	2.9	-	3.2	3.15	3.4	16	2000	-
[14]	-	-	-	2.5	-	2.5	3	3.4	12	1890	-
[11]	-	-	-	-	-	-	-	-	-	-	90
[14]	-	-	-	2.35	-	2.65	2.65	3	9	1760	-
[11]	-	-	-	-	-	-	-	-	-	-	90
[11]	-	-	-	-	-	-	-	-	-	-	90

### 3.2. Durability Characteristics of HDPE Fine Aggregate Concrete

Water adsorption of HDPE fine aggregate concrete has increased from 5% to 10.4%, when recycled HDPE content increases from 0% to 15% [15]. Similarly an increment of 5.5 kg/m^2^/min has been observed in initial rate of adsorption (IRA) from 0.5 kg/m^2^/min to 6.0 kg/m^2^/min, with the increase of HDPE from 0% to 15% [15]. It is observed that the chloride ion penetration is reduced and lies in the range of 2000–4000 Coulombs with the increase of HDPE content in concrete [12]. For instance, when HDPE increased from 0% to 15% in concrete, the chloride permeability has reduced from 4250 to 2700 Coulombs which is a 36.5% reduction [12].

After 28 days of curing, the Ultrasonic Pulse Velocity (UPV) test was performed on HDPE fine aggregate concrete and it is observed that the velocity has decreased from 3880 m/s to 2720 m/s, with the increase of HDPE from 0% to 60% in 15% intervals [14]. Additionally, slight drops of 6 m/s, 41 m/s, 19 m/s, 118 m/s and 170 m/s were observed in UPV at HDPE-0, HDPE-15, HDPE-30, HDPE-45 and HDPE-60, respectively, over the curing period of 28 to 90 days [14].

Thermal conductivity has dropped from 2 W/m·K to 1.14 W/m·K when HDPE increases from 0% to 60% at 7 days [14]. Similar readings have been observed in thermal conductivity variation for 14, 28, 90 and 365 days which are slight drops of 0.8 W/m·K, 0.81 W/m·K, 0.76 W/m·K and 0.69 W/m·K, respectively, with the increase of HDPE percentage in concrete [14]. It was also observed that thermal conductivity has dropped between 7 and 90 days, when HDPE is added from 0% to 60% in 15% intervals [14]. These reductions are recorded to be by 11%, 5.2%, 6.7%, 3.8% and 10.5% for HDPE-0, HDPE-15, HDPE-30, HDPE-45 and HDPE-60, respectively [14]. However, after 90 days, both standard and HDPE fine aggregate concretes have shown a stable conductivity as the decrease of thermal conductivity between 90 and 365 days are 2.8%, 3%, 0%, 4% and 1.9% for HDPE-0, HDPE-15, HDPE-30, HDPE-45 and HDPE-60 concrete, respectively [14].

### 3.3. Mechanical Properties of HDPE Coarse Aggregate Concrete

The workability (slump) of HDPE coarse aggregate concrete has reduced from 61 mm to 55 mm, and to 28 mm, when HDPE is increased from 0% to 4% in 2% intervals while keeping the water/cement (W/C) ratio at 0.55, Table 3 [16]. HDPE-6 and HDPE-8 showed zero slump with the same W/C ratio of 0.55 [16]. Another experiment has recorded 55 mm and 13 mm slumps at HDPE-0 and HDPE-100 concretes [17]. Both experiments have observed a reduction in the workability of the HDPE coarse aggregate concrete with the increase of HDPE. Philomina and D’Mello [18] have observed that the slump has increased from 95 mm to 118 mm, an increase of 24.2%, when HDPE is added from 0% to 32%. Similarly, it has also observed an increment in the slump from 10 mm to 18 mm with the increase of HDPE from 0% to 30% in 10% intervals [19].

**Figure 4:**
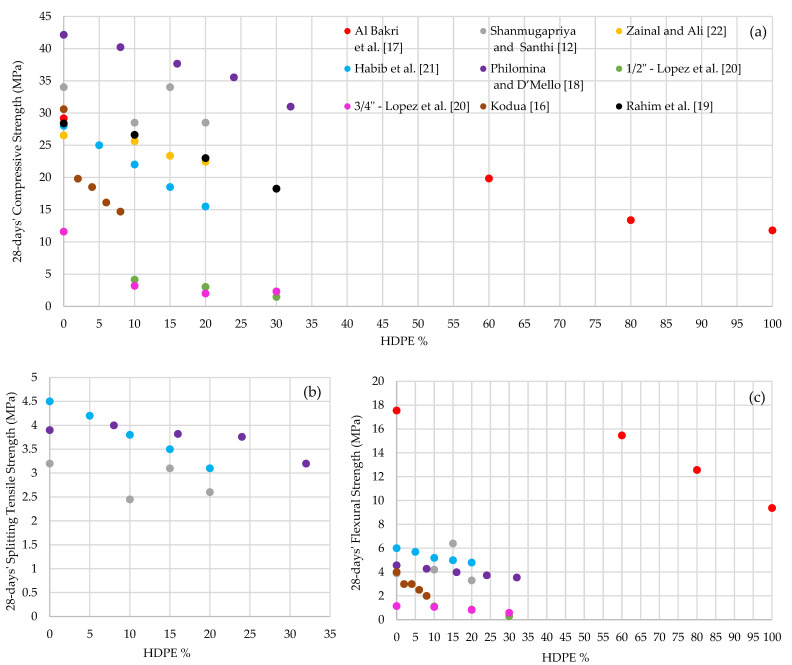
28-days’ (**a**) Compressive strength; (**b**) Splitting tensile strength and (**c**) Flexural strength variations of HDPE coarse aggregate concrete.

Similar to HDPE fine aggregate concrete, the compressive strength of HDPE coarse aggregate concrete decreases with the increases of HDPE percentage, Figure 4a. Lopez et al. [20] observed compressive strength reduction from 11.6 MPa to 2.3 MPa when HDPE is added from 0% to 30%. Authors further noted that the variation of HDPE coarse aggregate size between ½′′ and ¾′′ has not made any significant impact on the compressive strength [20]. Philomina and D’Mello [18] reported a compressive strength reduction from 42.14 MPa to 30.98 MPa with the increase of HDPE from 0% to 32% in 8% intervals. Rahim et al. [19] have observed that the 28-days’ compressive strength has dropped from 28.4 MPa to 18.24 MPa, when HDPE is added from 0% to 30% in 10% intervals, which is a drop from 28 MPa to 15.5 MPa by Habib et al. [21], when HDPE is increased from 0% to 20% in 5% intervals and a drop of 15.9 MPa by Kodua [16] with the increase of HDPE from 0% to 8% in 2% intervals. In another study, there is no compressive strength change noted up to 10% of HDPE incorporated in concrete, but afterwards, a little strength drop (i.e., from 26.54 MPa to 22.45 MPa) was observed when HDPE is added from 10% to 30% [22]. Similarly, the compressive strength has decreased from 34 MPa to 28.5 MPa with the increase of HDPE from 0% to 10%, an increment of 5.5 MPa during 10% to 15% increase and again, a drop of 5.5 MPa, when HDPE is increased from 15% to 20% [12]. It is identified that optimum 28-days’ compressive strength of 34 MPa is achieved at HDPE-15 [12]. However, Lopez et al. [20] suggest that optimum 28-days’ compressive strength is obtained at HDPE-10.

**Table 3:** Mechanical properties of HDPE coarse aggregate concrete.

References	Mix Design									Mechanical Properties											
	Cement (kg/m^3^)	HDPE %	Crushed Stone %	HDPE (kg/m^3^)	Crushed Stone (kg/m^3^)	Sand (kg/m^3^)	Water (kg/m^3^)	W/C Ratio	Admixtures/ Superplasticizer (w%)	Compressive Strength (MPa)				Split Tensile Strength (MPa)			Flexural Strength (MPa)			Density (kg/m^3^)	Slump (mm)
										7 days	14 days	28 days	56 days	7 days	14 days	28 days	7 days	14 days	28 days		
[17]	380	0	100	0	1045	665	190	0.5	-	-	-	29.19	-	-	-	-	-	-	17.56	-	55
[22]	-	0	100	-	-	-	-	0.55	-	21.32	23.68	26.54	-	-	-	-	-	-	-	-	-
[12]	320	0	100	0	1286.5	848.6	-	-	-	24	28	34	-	1.25	2.9	3.2	2.4	3.1	3.9	-	-
[21]	-	0	100	-	-	-	-	0.5	-	18.5	22	28	-	3.2	3.6	4.5	-	-	6	2365.74	-
[18]	-	0	100	-	-	-	-	0.45	X	23.71	30.5	42.14	43.61	2.87	3.3	3.9	-	-	4.56	2438.51	95
[20]	-	0	100	-	-	-	-	-	Y	11.59	-	-	-	-	-	-	-	-	1.15	1855.23	-
[16]	400	0	100	0	1200	800	-	0.55	-	13.9	19.5	30.6	-	-	-	-	-	-	4	-	61
[19]	-	0	100	-	-	-	-	0.55	-	20.236	25.482	28.402	-	-	-	-	-	-	-	-	10
[23]	-	0	100	-	-	-	-	0.4	-	-	-	10000 Nm/kg	-	-	-	-	-	-	-	-	-
[16]	400	2	98	24	1176	800	-	0.55	-	12.6	16.1	19.8	-	-	-	-	-	-	5	-	55
[16]	400	4	94	48	1152	800	-	0.55	-	12.5	14.1	18.5	-	-	-	-	-	-	3	-	28
[21]	-	5	95	-	-	-	-	0.5	-	17	19	25	-	2.8	3.4	4.2	-	-	5.7	2336.17	-
[16]	400	6	94	72	1128	800	-	0.55	-	11.3	13.6	16.1	-	-	-	-	-	-	2.5	-	0
[18]	-	8	92	-	-	-	-	0.45	X	22.5	26.3	40.23	45.32	2.64	3.56	4	-	-	4.28	2358.51	108
[16]	400	8	92	96	1104	800	-	0.55	-	9.4	10.3	14.7	-	-	-	-	-	-	2	-	0
[22]	-	10	90	-	-	-	-	-	-	18.24	22.35	25.64	-	-	-	-	-	-	-	-	-
[12]	320	10	90	128.68	1157.86	848.6	-	-	-	22	24	28.5	-	1.4	1.9	2.45	3.4	4	4.2	-	-
[21]	-	10	90	-	-	-	-	0.5	-	14	17	22	-	2.6	3.1	3.8	-	-	5.2	2302.52	-
[20]	-	(1/2 𠌼) 10	90	-	-	-	-	-	Y	4.147	-	-	-	-	-	-	-	-	1.117	1506.79	-
	-	(3/4 𠌼) 10	90	-	-	-	-	-		3.183	-	-	-	-	-	-	-	-	1.075	1466.88	-
[19]	-	10	90	-	-	-	-	0.55	-	18.964	22.706	26.617	-	-	-	-	-	-	-	-	12.7
[23]	-	10	90	-	-	-	-	0.4	-	-	-	4500 Nm/kg	-	-	-	-	-	-	-	-	-
[22]	-	15	85	-	-	-	-	-	-	17.23	20.49	23.37	-	-	-	-	-	-	-	-	-
[12]	320	15	85	192.99	1093.55	848.6	-	-	-	24.5	26.5	34	-	1.9	2.5	3.1	3.7	4.7	6.4	-	-
[21]	-	15	85	-	-	-	-	0.5	-	13	14	18.5	-	2.5	2.9	3.5	-	-	5	2256.63	-
[23]	-	15	85	-	-	-	-	0.4	-	-	-	3800 Nm/kg	-	-	-	-	-	-	-	-	-
[18]	-	16	84	-	-	-	-	0.45	X	21.18	24.73	37.66	43.86	1.96	2.92	3.82	-	-	3.98	2352.59	110
[22]	-	20	80	-	-	-	-	-	-	15.67	19.13	22.45	-	-	-	-	-	-	-	-	-
[12]	320	20	80	257.31	1029.23	848.6	-	-	-	21.5	23.5	28.5	-	1.7	2.2	2.6	-	-	-	-	-
[21]	-	20	80	-	-	-	-	0.5	-	11	13	15.5	-	2.4	2.6	3.1	-	-	4.8	2221.96	-
[20]	-	(1/2 𠌼) 20	80	-	-	-	-	-	Y	3	-	-	-	-	-	-	-	-	0.85	1324.89	-
	-	(3/4 𠌼) 20	80	-	-	-	-	-		2	-	-	-	-	-	-	-	-	0.72	1254.32	-
[19]	-	20	80	-	-	-	-	0.55	-	14.161	18.298	22.997	-	-	-	-	-	-	-	-	15.3
[23]	-	20	80	-	-	-	-	0.4	-	-	-	3000 Nm/kg	-	-	-	-	-	-	-	-	-
[18]	-	24	76	-	-	-	-	0.45	X	16.66	22.91	35.54	42.28	1.74	2.88	3.76	-	-	3.73	2348.14	115
[23]	-	25	75	-	-	-	-	0.4	-	-	-	2200 Nm/kg	-	-	-	-	-	-	-	-	-
[20]	-	(1/2 𠌼) 30	70	-	-	-	-	-	Y	1.445	-	-	-	-	-	-	-	-	0.29	1079.13	-
	-	(3/4 𠌼) 30	70	-	-	-	-	-		2.3	-	-	-	-	-	-	-	-	0.58	1179.51	-
[19]	-	30	70	-	-	-	-	0.55	-	10.835	14.037	18.244	-	-	-	-	-	-	-	-	18
[18]	-	32	68	-	-	-	-	0.45	X	14.84	20.46	30.98	39.85	1.58	2.62	3.2	-	-	3.54	2311.11	118
[17]	380	60	40	172	412	665	190	0.5	-	-	-	19.85	-	-	-	-	-	-	15.47	-	-
[17]	380	80	20	229	209	665	190	0.5	-	-	-	13.37	-	-	-	-	-	-	12.56	-	-
[17]	380	100	0	286	0	665	190	0.5	-	-	-	11.79	-	-	-	-	-	-	9.37	-	13

X = Conplast SP 430 G8; Y = 15% acrylic additive (PCHA).

The splitting tensile strength also shows a decreasing trend with the increment of HDPE content, Figure 4b [12,18,21]. A tensile strength reduction of 1.4 MPa (is observed at 28 days with the increase of HDPE from 0% to 20% [21]. There is no tensile strength change recorded until HDPE is added up to 8%, but 0.8 MPa strength reduction was noted when HDPE increased to 32% [18]. Shanmugapriya and Santhi [12] noted the contradicted behavior illustrating a significant splitting tensile strength reduction of 23.4% when incorporated with 10% HDPE in concrete. However, tensile strength gain (26.5%) was observed with the increase of HDPE content from 10% to 15% and, again, a drop of 16.1% when HDPE increased from 15% to 20% [12]. It is identified that HDPE-15 achieves the optimum 28-days’ splitting tensile strength.

Flexural strength or bending strength reduces with the increase of HDPE when incorporated as coarse aggregate in concrete Figure 4c. Bakri et al. [17] noted that the flexural strength decreased from 17.56 MPa to 15.47 MPa with the increase of HDPE content up to 60%. The flexural strength has dropped by 6.1 MPa when the HDPE is added from 60% to 100% [17]. It has also been observed that the 28-days’ flexural strength of HDPE coarse aggregate concrete is dropped by 1.9 MPa (from 6 MPa to 4.1 MPa) when HDPE increases from 0% to 20% in 5% intervals [21]. A 2 MPa flexural strength drop is observed with the increase of HDPE from 0% to 8% [16,18,20]. Shanmugapriya and Santhi [12] observed the opposite trend as a 2.5 MPa flexural strength gain obtained at 28 days for HDPE-15 concrete. The same experiment has achieved the optimum 28-days’ flexural strength of 6.4 MPa at HDPE-15, followed by a drop of 3.1 MPa, when HDPE is increased by another 5% [12]. It is noted that the optimum 28-days’ flexural strength is attained in different HDPE percentages: for instance, 6.4 MPa at HDPE-15 (Shanmugapriya and Santhi) [12], 4.28 MPa at HDPE-8 (Philomina and D’Mello) [18] and 1.075 MPa at HDPE-10 (Lopez et al.) [20].

The density of HDPE coarse aggregate concrete is decreased with the increase of HDPE content, Table 3 [18,20,21]. It is observed that there was a 5.2% reduction in density with the increase of HDPE from 0% to 32% and a 6.1% reduction during 0% to 20% of HDPE addition [12,21]. The density of ½′′ HDPE coarse aggregate concrete matrix has reduced by 776.1 kg/m^3^ and by 675.72 kg/m^3^ in ¾′′ HDPE coarse aggregate concrete matrix only with the increase of HDPE from 0% to 30% [20]. This reveals that a greater density drop could be achieved when the aggregate size is reduced from ¾′′ to ½′′ regardless of the HDPE inclusion percentage. Another notable observation is that the rate of reduction of dry density was 0.2% per volume percent of waste polymer added in HDPE coarse aggregate concrete [23].

### 3.4. Durability Characteristics of HDPE Coarse Aggregate Concrete

Porosity and permeability of HDPE coarse aggregate concrete have increased with the increase of HDPE content [20]. The HDPE-0 standard concrete displayed a porosity of 22.67 and increased it up to 36.21 when HDPE was added to 30% [20]. The typical value of permeability of a pervious concrete ranges from 0.135 to 1.219 cm/s and all the HDPE coarse aggregate concretes display a higher permeability than the range specified [20]. Permeability of ½′′ HDPE coarse aggregate concrete has increased by 5.28 cm/s, when the HDPE increases from 0% to 30% in 10% intervals [20]. Similarly, the permeability was increased by 4.03 cm/s, when ¾′′ HDPE coarse aggregates are increased in the same range [20]. It can be noted that the permeability of HDPE coarse aggregate concrete of this research study has exceeded the industry standards and varies between 0.135467 to 1.2192 cm/s [20]. Moreover, the porosity of HDPE coarse aggregate concrete has increased by 65% and 59.7%, with the increase of HDPE from 0% to 30% for ½′′ and ¾′′ aggregate sizes, respectively. An extensive study carried out on sorptivity of HDPE coarse aggregate concrete results in a drop of 45.4% in sorptivity with the increase of HDPE from 0% to 32% [18].

Water absorption rate of HDPE coarse aggregate concrete is increased by 34.7%, when the HDPE increase from 0% to 20% [22]. In another experiment, a similar gain, 35.5% in water absorption was observed with the increase of HDPE content to 8% [16]. However, Philomina and D’Mello [18] have observed a 6.13% drop (from 4.4% to 4.13%) in water absorption rate of HDPE coarse aggregate concrete when HDPE is added from 0% to 32% [18].

At direct UPV test, the pulse velocity of HDPE coarse aggregate concrete has increased from 4200 m/s to 4650 m/s, when HDPE is added from 0% to 16% [18]. After reaching the maximum velocity, 4650 m/s at HDPE-16, a drop of 560 m/s was recorded with the increase of HDPE from 16% to 32% [18]. The same experiment has performed an indirect UPV test for HDPE coarse aggregate concrete. Initially, the velocity is increased by 560 m/s, when HDPE increases from 0% to 8% [18]. Then, an 830 m/s velocity drop during 8% to 16% HDPE addition is observed, followed by a 1030 m/s velocity gain with the increase of HDPE from 16% to 24% and, finally, a drop of 1860 m/s, when HDPE is added from 24% to 32% [18]. The same study has carried out the rebound hammer test and have obtained rebound values at 56-days’ compressive strength [18]. The 56-day rebound values have increased from 40 to 42 when HDPE is added from 0% to 8% and then have dropped from 42 to 36 with the increase of HDPE from 8% to 32% [18]. Compressive strength obtained through rebound hammer test as well as through a destructive test show similar increments by 2.1 MPa and 1.71 MPa when HDPE increases from 0% to 8% and drops by 6.32 MPa and 5.47 MPa when HDPE is added from 8% to 32%, respectively [18].

When considering the sulphate attack test, the compressive strength of HDPE coarse aggregate concrete (with the increase of HDPE from 0% to 32%) is increased when immersed with Na2SO4 apart from the standard concrete matrix (HDPE-0) [18]. Compressive strength is observed to be dropped by 1.48 MPa in the standard concrete matrix (HDPE-0) after immersion in Na2SO4 [18]. Compressive strength is increased by 0.33 MPa, 0.51 MPa, 0.58 MPa and 1.63 MPa at HDPE-8, HDPE-16, HDPE-24 and HDPE-32 concretes, respectively [18]. This has further displayed a 6.25% of weight loss due to a sulphate attack test in HDPE coarse aggregate concrete with the increase of HDPE from 0% to 32% [18].

An acid attack test was carried out through a comparison of compressive strength values obtained after immersing HDPE coarse aggregate concrete mixes in both water and hydrochloric (HCl) acid after curing for 28 days [18]. When the HDPE is increased from 0% to 32% in 8% intervals, the compressive strength has reduced by 12.21 MPa and 12.45 MPa after immersion in HCl acid and water, respectively. When comparing the compressive strength drop of each HDPE coarse aggregate concrete matrix: HDPE-0, HDPE-8, HDPE-16, HDPE-24 and HDPE-32 due to immersion in HCl acid is recorded to be 2.7%, 5.0%, 4.1%, 3.31% and 2.97%, respectively [18]. The least compressive strength drop was recorded in HDPE-0 concrete matrix and, however, it can be observed that the compressive strength drop has reduced significantly with an increase of HDPE. The same experiment has observed an increase of 54.5% in weight loss percentage, with the increase of HDPE from 0% to 32% [18]. According to Kodua [16], when concrete mixes are immersed in nitric (HNO3) acid solution, compressive strength of HDPE coarse aggregate concrete is decreased by 53.9% with the increase of HDPE from 0% to 8%. An increase in weight loss of 34.1% was also observed, after immersing in HNO3, when HDPE is increased from 0% to 8% [16]. Therefore, the effects imposed due to acids on HDPE coarse aggregate concrete are very minimal and can withstand the chemical reactions within the concrete [18].

## 4. Use of HDPE as a Fiber Reinforcement in Concrete

### 4.1. Mechanical Properties of HDPE Fiber Reinforced Concrete

Table 4 illustrates that the workability (slump) of HDPE fiber reinforced concrete was reduced by 73.8% when adding 1.25% of HDPE fibers having a diameter of 0.25 mm and a length of 23 mm while maintaining the same w/c ratio of 0.62 [24,25]. Pešić et al. [24] reveal that varying the fiber diameter from 0.25 mm to 0.4 mm and length from 23 mm to 30 mm, has reduced the slump by 3 mm at HDPE-0.4 and by 4 mm at HDPE-0.75 and HDPE-1.25. It also suggests that increasing the water content used in HDPE concrete matrix and adding water-reducing agents can prevent segregation of plastic fibers in the reinforced concrete mixes [26]. As a result, Dehydol LS-12 (LS-12) nonionic surfactant is used to increase the wettability of plastic materials [26]. With the addition of the Dehydol LS-12, it was observed that the slump values are decreased from 130 mm to 20 mm when w/c ratio is increased from 0.5 to 0.6 and the HDPE fiber are increased from 0% to 30% [26].

**Figure 5:**
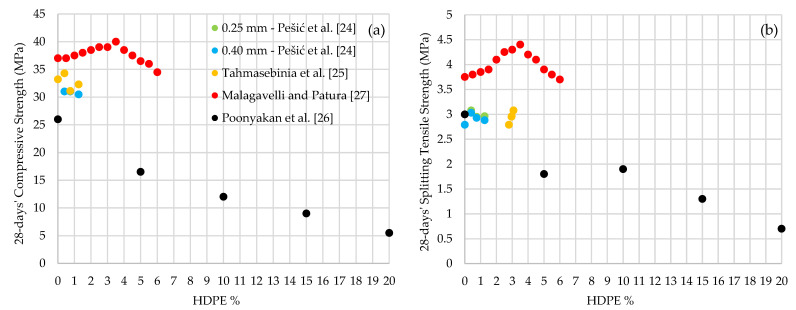
28-days’ (**a**) Compressive strength and (**b**) Split tensile strength variations of HDPE fiber reinforced concrete.

As illustrated in Figure 5a, the compressive strength has decreased by 8.13% when HDPE fibers with a diameter of 0.4 mm are added from 0% to 1.25% [24]. Moreover, when the HDPE fiber diameter is 0.25 mm, the compressive strength has increased by 3.3% with the increase of HDPE fibers from 0% to 0.4% and, again, dropped by 5.8% when fibers are added from 0.4% to 1.25% [24,25]. Malagavelli and Patura [27] showed that the optimum compressive strength of 40 MPa was achieved by HDPE-3.5 concrete when the fiber content is varied between 0% and 6%. When the fibers are added by 5% intervals from 0% to 20%, the compressive strength has significantly dropped from 26 MPa to 20 MPa [26]. However, it has been identified that the large volume fraction of HDPE fibers often lowers the compressive strength of concrete [26]. Poonyakan et al. [26] have suggested that all concrete used for precast wall panels (non-structural load carrying) must have a minimum compressive strength of 16 MPa which can be fulfilled by adding HDPE fibers up to 5% [26].

The 28-days’ splitting tensile strength of HDPE fiber reinforced concrete has increased by 10.4%, when fibers with 0.25 mm diameter are added from 0% to 0.4% and again a 3.9% drop is observed, when fibers are added from 0.4% to 1.25%, Figure 5b [24,25]. Similarly, the splitting tensile strength has increased from 2.79 MPa to 3.03 MPa, when fibers with 0.40 mm diameter are added from 0% to 0.4%, and a drop of 0.15 MPa is observed, when HDPE fibers are added from 0.4% to 1.25% [24]. Similar to compressive strength observations, Malagavelli and Patura [27] have observed the optimum splitting tensile strength of 4.4 MPa at HDPE-3.5, when the fiber content varied between 0% and 6%. In another experiment, the splitting tensile strength has dropped by 40% with the increase of HDPE from 0% to 5%, followed by a slight gain of 5.6% at HDPE-10 and then, again, tensile strength has dropped by 63.2%, when HDPE fibers are added from 10% to 20% [26]. On the other hand, the 28-days’ flexural strength of HDPE fiber reinforced concrete is always greater than the standard concrete (HDPE-0) regardless of the HDPE fiber percentage being added, Table 4 [27]. It is observed that, the flexural strength has increased from 3.4 MPa to an optimum of 4.9 MPa when the HDPE fibers are added from 0% to 3% in 0.5% intervals and then the flexural strength has dropped by 1.3 MPa when the HDPE fibers are added from 3% to 6% in 0.5% intervals [27].

A slight increase (1.0 GPa) is observed in the elastic modulus of HDPE fiber reinforced concrete with the increase of 0.25 mm diameter HDPE fibers from 0% to 1.25%, Table 4 [24]. The same experiment shows that the elastic modulus has increased by 1.3 GPa, when the 0.4 mm diameter HDPE fibers are added from 0% to 1.25% [24].

### 4.2. Durability Characteristics of HDPE Fiber Reinforced Concrete

When considering the water permeability after 45 days, it has been identified that, the height of water penetration in HDPE fiber reinforced concrete has reduced from 43 mm to 26 mm, when HDPE fibers with 0.4 mm diameter are added from 0% to 1.25% [24]. Similarly, HDPE fibers with 0.25 mm diameter have reduced the height of water penetration from 43 mm to 28 mm in similar HDPE percentages [24]. HDPE fiber reinforced concrete has contributed in reducing the water intake from 35% to 80% with the increase of HDPE fibers from 0.40% to 1.25% compared to HDPE-0 standard concrete [24]. Poonyakan et al. [26] have observed that the porosity of the HDPE fiber reinforced concrete has increased by 45% when HDPE is added from 0% to 30%. Hence, it has been identified that the recycled HDPE fibers greatly improve the durability of concrete.

It has been observed that the overall number of cracks and the widths of cracks were reduced in HDPE fiber reinforced concrete when compared with the standard concrete [24]. Average crack width and the maximum crack width of 0.25 mm HDPE fiber reinforced concrete have been reduced by 83.63% and 81.8%, respectively when HDPE fibers are added from 0% to 1.25% [24]. Similarly, average crack width and the maximum crack width of 0.4 mm HDPE fiber reinforced concrete have been reduced by 76.4% and 77.3% when HDPE fibers are added from 0% to 1.25% [24]. Crack reduction ratio of 0.25 mm and 0.4 mm HDPE fiber reinforced concrete increased from 34.5% to 83.6%, and from 54.5% to 76.4% when HDPE fibers are increased from 0.4% to 1.25%, compared with the standard concrete matrix (HDPE-0) [24].

## 5. Use of HDPE as a Cement Binder

Aattache et al. [28] investigated the cement replacement using HDPE up to 6%. It is observed that the 28-days’ compressive strength has decreased from 37 MPa to 22 MPa, which is a 40.5% drop, when HDPE is added from 0% to 6% [28]. Similarly, the 7-day and 90-day compressive strength variations display decreasing trends with a strength drop of 46.7% and 28.6%, respectively, with the increase of similar HDPE percentages [28]. Similar to compressive strength, the splitting tensile strength also decreases with the increase of HDPE from 0% to 6% in 2% intervals over 7, 28 and 90 days [28]. They have observed tensile strength decrease of 1.7 MPa, 3.9 MPa and 3.2 MPa at 7, 28 and 90 days, respectively, with the use of HDPE in 6% [28].

HDPE has a comparatively low thermal conductivity of 0.33 W/m·K compared with the concrete [28]. It was observed that the thermal conductivity of cement replaced HDPE concrete is decreased with the increase of HDPE [28]. At 20 °C, the 28-days’ thermal conductivity of cement replaced HDPE concrete is decreased by 15.6%, when HDPE is increased from 0% to 6% in 2% intervals [28]. Similar observations are recorded for 28-days’ thermal conductivity for 140 °C, 250 °C and 350 °C which reduce by 15.8%, 22.2% and 12%, respectively. Thermal conductivity of cement replaced HDPE concrete is decreased with an increase of temperature from 20 °C to 350 °C, when HDPE is added up to 6% [28]. It was also observed that concrete mixes with a superplasticizer have a higher thermal conductivity in comparison with the standard concrete matrix (HDPE-0) since the 28th day [28].

On the other hand, Naik et al. [29] investigated the use of HDPE as a filler material in concrete, where HDPE is added up to 2% in 0.5% intervals. It was observed that the 28-days’ compressive strength of HDPE filler concrete was increased by 2.5%, when HDPE is added from 0% to 0.5% [29]. Then, a drop of 46.3% has been observed in compressive strength with the increase of HDPE from 0.5% to 2% in 0.5% intervals [29]. Similar gains of 3.2% and 13% were observed for 3-day and 7-day compressive strengths, respectively, when HDPE is increased from 0% to 0.5% [29]. Additionally, 25% and 42.3% drops in 3-day and 7-day compressive strength were observed when HDPE increased from 0.5% to 2% [29].

## 6. Discussion

The aggregate shape, size, and surface texture influence the concrete workability and the bond between aggregates and cement/binder matrix, which in turn impact the mechanical performance in concrete [17]. Slump of HDPE coarse aggregate concrete is varied between 0 mm and 25 mm with the increase of HDPE percentage. This is due to the fact that the low workability in the concrete matrix is a result of some HDPE particles being angular and the rest having non-uniform shapes [19]. These different and non-uniform shaped HDPE particles considerably fail in filling the voids with HDPE fine and coarse aggregates and obstruct the flow of the concrete mix, with the increase of HDPE fine and coarse aggregates [16,19]. The reason for the reduction of density in HDPE aggregate concrete with the increase of HDPE is mainly due to the difference in the densities of HDPE and sand and the lower unit weight of HDPE [14,23] The density of sand and HDPE is 1600 kg/m3 and 950 kg/m3, respectively, which is HDPE being about 40% lighter than the sand [14]. Therefore, the overall density of the HDPE fine aggregate concrete is decreased with the increase of HDPE content in concrete [15].

**Figure 6:**
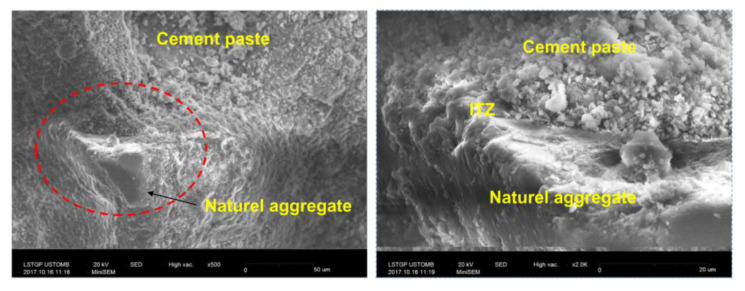
SEM images with weaker ITZ interfaces of HDPE-60 concrete matrix [14].

**Figure 7:**
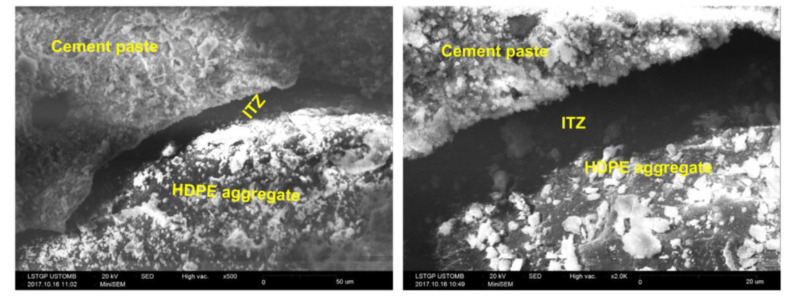
SEM images with stronger ITZ interfaces of standard concrete matrix (HDPE-0) [14].

There are various techniques used to evaluate the performance of concrete. Scanning Electron Microscopy (SEM) images are used to examine the microstructure development of the HDPE incorporated concrete. Similarly, nanoindentation is used to observe the interfacial transition zone (ITZ) of the concrete matrix and Computed Tomography (CT) to identify the pore-structure of the concrete. Poor bond capacity and the availability of higher air content in the concrete matrix with the inclusion of HDPE aggregates is due to the non-reactive (chemically inert) behavior that leads to reduced mechanical and durability performance of HDPE aggregate concrete [30]. Weaker bonds and lower adhesion strength between cement paste and the HDPE aggregates have occurred mainly due to the hydrophobic nature and the smooth and glossy surface texture of the HDPE particles in rounded shape than a crushed aggregate [14,15,17]. Hydrophobic nature of HDPE results in weaker bonds with lesser strength between cement/binder matrix and HDPE aggregates at the interfacial transition zone (ITZ) when compared with the ITZ formed between natural fine aggregates (sand) and cement paste, Figure 6 and Figure 7 [14]. These weaker bonds have resulted in reducing the compressive and tensile strengths and the modulus of elasticity of both HDPE fine and coarse aggregate concretes with the increase of recycled HDPE percentage [12,14,19,22,23]. Apart from the weaker bonds between cement paste and HDPE particles, hydrophobic effect further limits the inhibition of the cement hydration by restricting the movement of water, and may lead to reduce both durability and mechanical performances of HDPE fine and coarse aggregate concrete with the increase of HDPE [30]. Additionally, higher porosity and large air bubbles entrapped in the HDPE fine and coarse aggregate concrete can result in reducing the compressive strength with the increase of HDPE [10,12,14,15]. This ductile behavior in HDPE can significantly reduce crack formation and propagation in concrete as the cracks are being trapped by the HDPE particles [12]. HDPE aggregate concrete showed higher tensile strength than standard concrete (HDPE-0) that may be associated with the ductility of HDPE fine and coarse aggregates [12]. It was also observed that when HDPE fine aggregate concrete samples are broken, that most HDPE particles in the concrete matrix have not been broken; instead they had peeled off from the dry concrete matrix, when the breaking stress is reached [14]. Elastic modulus of a concrete mix is mainly based on the types of aggregates used, density and the compressive strength of the concrete [14]. Modulus of elasticity of HDPE coarse aggregate concrete decreases with the increase of HDPE which may be due to reasons such as increase of volume of pores in concrete matrix, the difference in modulus of elasticity values of HDPE particles and the concrete mix and internal defects (cracks occurred around HDPE particles) [14].

Increase in water adsorption of HDPE fine aggregate concrete with the increase of HDPE percentage is mainly due to the lack of interface bonding between HDPE and the aggregates, the increase in the porosity of the concrete matrix, and the higher HDPE content which occupies the space in the concrete, which could evaporate water which leads to leaving voids thus increasing the absorption value while creating more spaces to fill up with water [15,16]. The velocity of waves strongly depends on the mechanical properties of the concrete and is highly influenced by the voids in the concrete. The pores created because of HDPE particles slow down the velocity of the ultrasonic wave due to the increase in the acoustic impedance. When the incident wave passes through concrete, HDPE and pores, some waves are partially reflected, and the rest are transmitted. This phenomena leads to a reduction in wave velocity with the increase of HDPE content [14]. Thermal conductivity of HDPE fine aggregate concrete has dropped with the increase of HDPE content due to the storage of a significant amount of water in the pores [14]. It was also noted that the thermal conductivity of standard concrete (HDPE-0) is higher than that of the HDPE fine aggregate concrete which may be due to HDPE having a low thermal conductivity (0.4 W.m1 K1) compared with the conductivity of natural sand [14]. When considering the chloride ion penetration test, it was observed that chloride ion penetration has reduced and lies in the favorable range of 2000–4000 coulombs with the increase of HDPE mainly due to the impervious HDPE granules which block the passage of chloride ion penetration and deterioration of the concrete [12]. Testing the concrete mixes against chemical attacks is vital as the reaction of chemical elements from exposure and moisture present in the concrete can result in deterioration of concrete structures. Philomina and D’Mello [18] have noted that the effect of chemicals on HDPE concrete is lesser when compared with the standard concrete matrix (HDPE-0). They have further identified that the chemical composition of HDPE coarse aggregate concrete is having the ability to resist the chemical actions and corrosion [18]. When considering the acid attack test, the reduction in compressive strength and the weight of HDPE coarse aggregate concrete with the increase of HDPE is greater in H2SO4 acidic medium than the water [16]. This reduction in compressive strength and weight is due to the deposit of the gypsum which is formed following the action between the portlandite and H2SO4 acid [16].

The addition of HDPE and other plastic fibers to concrete mixes have shown more ductile behavior than conventional concrete [21]. According to Poonyakan et al. [26], the slump values are dropped with the increase of HDPE, resulting in a reduction in the workability of HDPE fiber reinforced concrete. This may lead to segregate the concrete due to the restriction of the movement of fresh concrete. The reduction of workability is due to the increase of the viscosity of fiber reinforced concrete and the improvement in absorption capacity of the cement paste with the increase of surface area of HDPE fibers at high volume fractions [26]. Compressive strength of fiber reinforced concrete decreases with the increase of HDPE fibers in large volume fractions, which may be due to the hydrophobic property of HDPE which is likely to increase air voids in the concrete matrix [26]. When ductile fibers are added into HDPE fiber reinforced concrete, it will improve its tensile strength depending on several factors such as fiber toughness, fiber volume fraction, alignment, and the adhesion strength between the fibers and the cementitious matrix [26]. Larger volume fractions of HDPE often leads to clumping or balling of fibers, making them less effective in strengthening the concrete composites [26]. The use of HDPE as a reinforcement material in concrete resulted in reducing the bulk density of produced HDPE fiber reinforced concrete than that of the standard concrete (HDPE-0), which may be due to the complex network structures created by HDPE which resulted in honeycombs [26]. Thermal conductivity of HDPE fiber reinforced concrete is not only affected by HDPE fiber properties, but also small permeable voids formed in the concrete mix which can also restrain heat transfer. It was observed that more permeable voids are formed in the HDPE fiber reinforced concrete with the increase of HDPE content, which will lead to a decrease in the thermal-inducing properties or heat transfer characteristics of concrete matrix [26].

**Figure 8:**
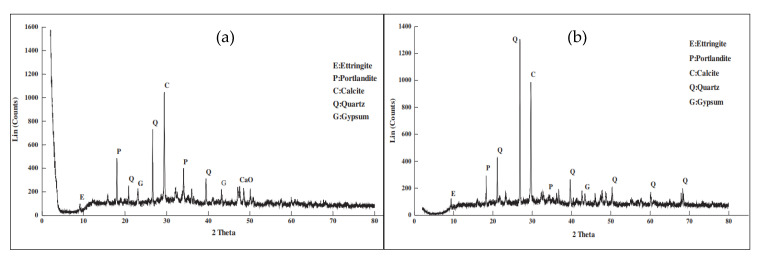
XDM spectrum of (a) Standard concrete Matrix (HDPE-0) and (b) Cement replaced HDPE-6 concrete matrix at 20 °C [29].

X-ray Diffract Meter (XDM) spectra analysis [31] was conducted by Aattache et al. [28] to identify the chemical interaction between the cement material and the HDPE fibers replaced. Figure 8a shows the XDM spectrum of standard concrete matrix (HDPE-0) at 20 °C which reveals that there is no chemical interaction between the cement and the HDPE and no formation of new chemical products. It also displays that a rearrangement of the crystalline structure has occurred during cement hydration, as absorption bands characterizing anhydrous clinker are replaced by the hydration particles [28]. Figure 8b further illustrates the XDM spectrum of cement replaced at 6-HDPE at 20 °C. It also reveals that there is no chemical interaction between the cement and the HDPE as well as no chemical products’ formation. However, it highlights the presence of high and intensive peaks of quartz, characterizing the presence of silica due to the adjuvant incorporated [28]. Similarly, the portlandite peak of standard motor without HDPE (18,092 h) is slightly greater than that of HDPE-6 because of the high rate of silica (90%) relative to portlandite, and the addition of HDPE may cause a progressive decrease of portlandite in the HDPE-6 relative to standard concrete [28]. Cement and HDPE morphologies have been observed under different temperatures and it was observed that with the increase of temperature, HDPE concrete becomes less compact and deteriorates. This phenomenon is clearly marked by the existence of pores, 108 µm in size at 250 °C and of 162 µm at 350 °C. The cracks were formed because of the absence of HDPE, letting the pores become the entry points for air [28]. Thermal conductivity of concrete where HDPE is used as a cement replacement was decreased with the increment of HDPE content due to the presence of pores distributed within the material in the concrete mix during the elimination of HDPE, reduction in pore appearance by nano-silicates, and as nano-particles behave as a filling agent [28].

## 7. Summary and Conclusions

The mechanical and durability properties of concrete incorporated with recycled HDPE as a fine aggregate, a coarse aggregate, a fiber, and a cement binder is comprehensively reviewed. The principal conclusions drawn are listed below:

- Optimum 28-days’ compressive and flexural strength of HDPE fine aggregate concrete is observed at HDPE-10 and splitting tensile strength at HDPE-5 whereas for HDPE coarse aggregate concrete, optimum compressive and flexural strength are recorded in the range of 10% to 15% of HDPE incorporation and splitting tensile strength at HDPE-15.
- Water absorption of HDPE fine and coarse aggregate concrete is increased by 5.4% and 35% with the increase of HDPE from 0% to 15% and 20%, respectively, while porosity and permeability of HDPE coarse aggregate concrete was increased by 65% and 461% with the increase of HDPE percentage from 0% to 30%.
- Similarly, other durability properties of HDPE fine and coarse aggregate concrete such as lower thermal conductivity (25% drop) and higher resistance against chloride, sulphate and acid attacks were observed with inclusion of HDPE up to 32% more than the standard/plain concrete matrix (HDPE-0) always.
- Increasing the HDPE percentage up to 1.25% and maintaining the w/c ratio at 0.62 reduces the workability by 80% and increases the elastic of modulus by 4.1% of HDPE fiber reinforced concrete.
- The 28-days’ flexural and splitting tensile strength of HDPE fiber reinforced concrete is increased up to an optimum of 4.9 MPa (at HDPE-3) and 4.4 MPa (at HDPE-3.5) with an increase of HDPE from 0% to 6%, and was higher than the standard/plain concrete matrix (HDPE-0) in all inclusion levels of HDPE.
- HDPE fiber reinforced concrete where HDPE is increased up to 1.25% display better durability properties than the standard/plain concrete matrix (HDPE-0) due to the reduction of water permeability by 39.5% and plastic shrinkage cracking by 83.6% when considering the average crack width reduction.
- Weaker bonds and lower adhesion strength between the concrete mix and HDPE aggregates are mainly due to the hydrophobic nature, smooth and glossy surface texture and the chemical inert behavior of HDPE which result in reducing both durability and mechanical performances of HDPE fine and coarse aggregate concrete with the increase of HDPE.
- When ductile fibers are added into HDPE fiber reinforced concrete, it will improve its tensile strength depending on several factors such as fiber toughness, fiber volume fraction, alignment, and the adhesion strength between the fibers and the cementitious matrix.

## Figures and Tables

**Table 4:** Mechanical properties of HDPE fiber reinforced concrete.

Reference	Mix Design									Mechanical Properties												
	Cement (kg/m^3^)	HDPE			HDPE (kg/m^3^)	Crushed Stone (kg/m^3^)	Sand (kg/m^3^)	Water Content (kg/m^3^)	W/C Ratio	Compressive Strength (MPa)			Split Tensile Strength (MPa)			Flexural Strength (MPa)			Modulus of Elasticity (GPa)		Bulk Density (kg/m^3^)	Slump (mm)
		L (mm)	d (mm)	%						7 days	28 days	90 days	7 days	28 days	90 days	7 days	28 days					
[24]	380	-	-	0	0	860	780	235	0.62	-	33.2	38.1	-	2.79	3.32	-	-		24.2		-	65
[25]	-	-	-	0	0	-	-	-	-	-	33.2	38.1	-	2.79	3.32	-	-		24.2		-	65
[27]	-	-	-	0	0	-	-	-	0.42	25	37	-	3	3.75	-	1.7	3.4		-		-	-
[26]	33 kg	-	-	0	0	-	66 kg	16.5 kg	0.5	-	26	-	-	3	-	-	-		-		1950	130
[24]	380	23	0.25	0.4	-	860	780	235	0.62	-	34.3	40.1	-	3.08	3.47	-	-		24.5		-	36
[24]	380	30	0.4	0.4	-	860	780	235	0.62	-	31	37.2	-	3.03	3.4	-	-		24.2		-	33
[25]	-	23	0.25	0.4	-	-	-	-	-	-	34.3	40.1	-	3.08	3.47	-	-		24.5		-	36
[27]	-	-	-	0.5	-	-	-	-	-	29	37	-	2.9	3.8	-	1.75	3.45		-		-	-
[24]	380	23	0.25	0.75	-	860	780	235	0.62	-	31.1	38.4	-	2.95	3.49	-	-		24.9		-	22
[24]	380	30	0.4	0.75	-	860	780	235	0.62	-	31	37.7	-	2.93	3.47	-	-		25.9		-	18
[25]	-	23	0.25	0.75	-	-	-	-	-	-	31.1	38.4	-	2.95	3.49	-	-		24.9		-	22
[27]	-	-	-	1	-	-	-	-	-	29.5	37.5	-	3.2	3.85	-	1.9	3.45		-		-	-
[24]	380	23	0.25	1.25	-	860	780	235	0.62	-	32.3	37.7	-	2.96	3.43	-	-		25.2		-	17
[24]	380	30	0.4	1.25	-	860	780	235	0.62	-	30.5	38.7	-	2.88	3.53	-	-		25.5		-	13
[25]	-	23	0.25	1.25	-	-	-	-	-	-	32.3	37.7	-	2.96	3.43	-	-		25.2		-	17
[27]	-	-	-	1.5	-	-	-	-	-	30	38	-	3.3	3.9	-	2	3.6		-		-	-
[27]	-	-	-	2	-	-	-	-	-	30.5	38.5	-	3.4	4.1	-	2.1	3.65		-		-	-
[27]	-	-	-	2.5	-	-	-	-	-	31	39	-	2.4	4.25	-	2.1	3.8		-		-	-
[27]	-	-	-	3	-	-	-	-	-	32	39	-	3.6	4.3	-	2.2	4.9		-		-	-
[27]	-	-	-	3.5	-	-	-	-	-	33	40	-	3.7	4.4	-	2.25	4.2		-		-	-
[27]	-	-	-	4	-	-	-	-	-	30.5	38.5	-	3.6	4.2	-	2.2	4		-		-	-
[27]	-	-	-	4.5	-	-	-	-	-	30	37.5	-	3.55	4.1	-	2.1	3.9		-		-	-
[27]	-	-	-	5	-	-	-	-	-	29.5	36.5	-	3.5	3.9	-	2.05	3.8		-		-	-
[26]	30.12 kg	-	-	5	0.14	-	60.24 kg	15.06	0.5	-	16.5	-	-	1.8	-	-	-		-		-	-
[27]	-	-	-	5.5	-	-		-	-	29	36	-	3.4	3.8	-	2	3.7		-		-	-
[27]	-	-	-	6	-	-		-	-	27.5	34.5	-	3.3	3.7	-	1.9	3.6		-		-	-
[26]	30.12 kg	-	-	10	0.28	-	60.24 kg	15.05kg	0.5	-	12	-	-	1.9	-	-	-		-		1750	60
[26]	30.12 kg	-	-	15	0.42	-	60.24 kg	16.57 kg	0.55	-	9	-	-	1.3	-	-	-		-		-	-
[26]	30.12 kg	-	-	20	0.56	-	60.24 kg	18.07 kg	0.6	-	5.5	-	-	0.7	-	-		-		-	1800	48
[26]	30.12 kg	-	-	30	0.84	-	60.24 kg	18.07 kg	0.6	-	-	-	-	-	-	-		-		-	1677	20

## Data Availability

All data are included in the manuscript
